# Rhinofacial entomophthoramycosis case series, the unusual cause of facial swelling

**DOI:** 10.1016/j.amsu.2020.07.013

**Published:** 2020-07-15

**Authors:** Saisawat Chaiyasate, Parichat Salee, Kornkanok Sukapan, Tanyathorn Teeranoraseth, Kannika Roongrotwattanasiri

**Affiliations:** a Department of Otolaryngology, Faculty of Medicine, Chiang Mai University, Chiang Mai, 50200, Thailand; b Department of Internal Medicine, Faculty of Medicine, Chiang Mai University, Chiang Mai, 50200, Thailand; c Department of Pathology, Faculty of Medicine, Chiang Mai University, Chiang Mai, 50200, Thailand

**Keywords:** Conidiobolus, Entomophthora, Zygomycosis

## Abstract

**Background:**

Rhinofacial entomophthoramycosis is a specific fungal infection of the skin and subcutaneous tissue. It is considered as a rare and neglected disease in tropical and subtropical areas. We would like to present our cases to aid other physicians in the improved recognition of typical cases.

**Materials and methods:**

A retrospective review was performed on patients with the diagnosis of Conidiobolomycosis or Entomophthoramycosis in Chiang Mai University Hospital, Thailand, from January 2009 to May 2019. There were seven cases with a definite pathologic report or culture in this review.

**Results:**

All seven patients were men and were referred to the university hospital for diagnosis. The mean age was 53 ± 15.7, ranging from 27 to 71 years. Most of the patients (85.7%) presented first with nasal or rhinofacial swelling and nasal obstruction. The definite diagnosis came from clinical presentation and investigation with a tissue biopsy, culture and communication among physicians. Patients responded well with a combination of medical treatment, including potassium iodide (KI), co-trimoxazole, or itraconazole.

**Conclusion:**

Rhinofacial entomophthoromycosis or Conidiobolomycosis typically can be diagnosed under a suspicious clinical presentation. The obvious clinical response can be seen within several weeks after medication.

## Highlights

• Rhinofacial entomophthoramycosis or Conidiobolomycosis is uncommon disease, but commonly presented with facial swelling.
• Typical clinical presentations were demonstrated to help other physicians in the improved recognition and diagnosis.
• This is a disease that once known, will not be forgotten.

## 1 Introduction

Rhinofacial entomophthoramycosis is a specific fungal infection of the skin and subcutaneous tissue. It is considered a rare and not well-known condition. The reported cases come from different specialties, such as dermatology, infectious-internal medicine, pathology and otorhinolaryngology and from tropical and subtropical countries [[1], [2], [3]]. Typical presentations are a progressive nasal obstruction and/or rhinofacial swelling.Rhinofacial entomophthoramycosis is caused by *Conidiobolus spp.*, while *Basidiobolus spp*. usually involves the extremities.

From the Medpilot database, Blumentrath et al. reviewed 198 cases from 117 records [3]. Then 145 cases and one added case were selected for analysis. The number of cases shows that this condition is not that rare but may be unknown by the physicians who encounter it. Now we are living in a connected world, where people can reach out to the other parts of the world easily using various kinds of transportation. Tropical diseases previously found in Africa, Asia, or South America can be seen in any part of the world. This study presents seven cases to help other physicians in the improved recognition of typical cases of Conidiobolomycosis.

## 2 Material and methods

A retrospective review was performed on patients with the diagnosis of Conidiobolomycosis or Entomophthoramycosis in a tertiary University Hospital from January 2009 to May 2019. Nine suspected cases were reviewed with the exclusion of two cases; a four-year-old boy who presented with lesions on his extremities and a 50-year-old man from a neighboring country who did not have a definite pathologic report. The tissue biopsy was performed under local or general anesthesia by otolaryngologists with no other specialized preoperative or perioperative intervention. Tissue pieces were sized more than 0.5 cm. Bleeding sites were cauterized or controlled with anterior nasal packing. Tissue was sent for pathologic report and, in some cases, fungal culture. There were seven cases in this review. This study was approved by the Research Ethics Committee of the university and written consent forms were obtained for patients’ photography presentation. The study was registered on the Thai Clinical Trials Registry (TCTR20200608006). This case series was reported in line with the PROCESS 2018 criteria [4].

## 3 Results

**Fig. 1:**
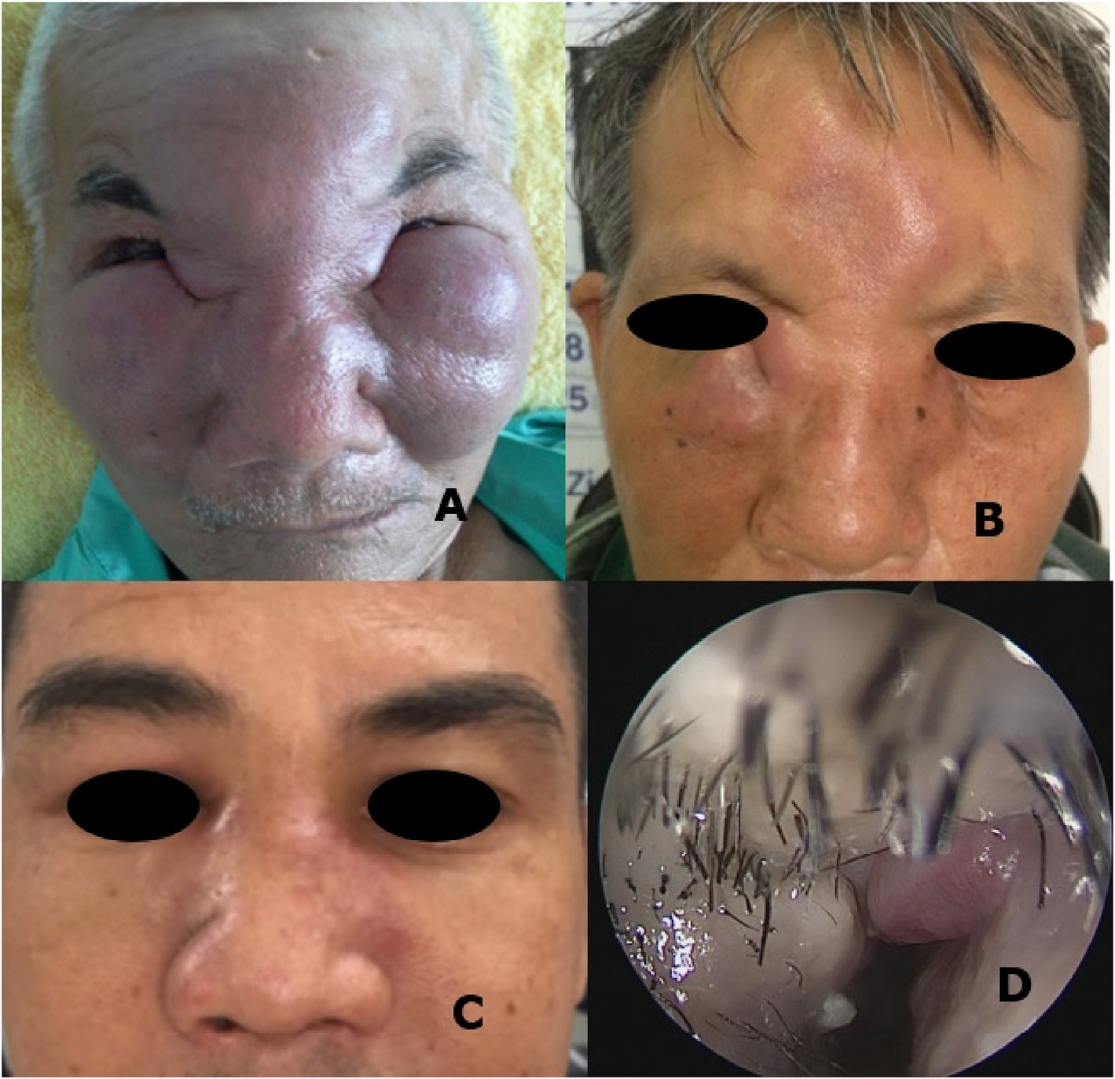
Erythematous skin A, B Firm indurated skin and subcutaneous tissue over cheeks, glabella and nasal dorsum (patients No.2 and 3) C, D Infiltrating lesion over nasal dorsum and in the nasal cavity, patient No. 5.

All seven patients were men. The mean age was 53 ± 15.7, ranging from 27 to 71 years. Most of the patients (85.7%) presented first with nasal or rhinofacial swelling and nasal obstruction (Fig. 1).

**Fig. 2:**
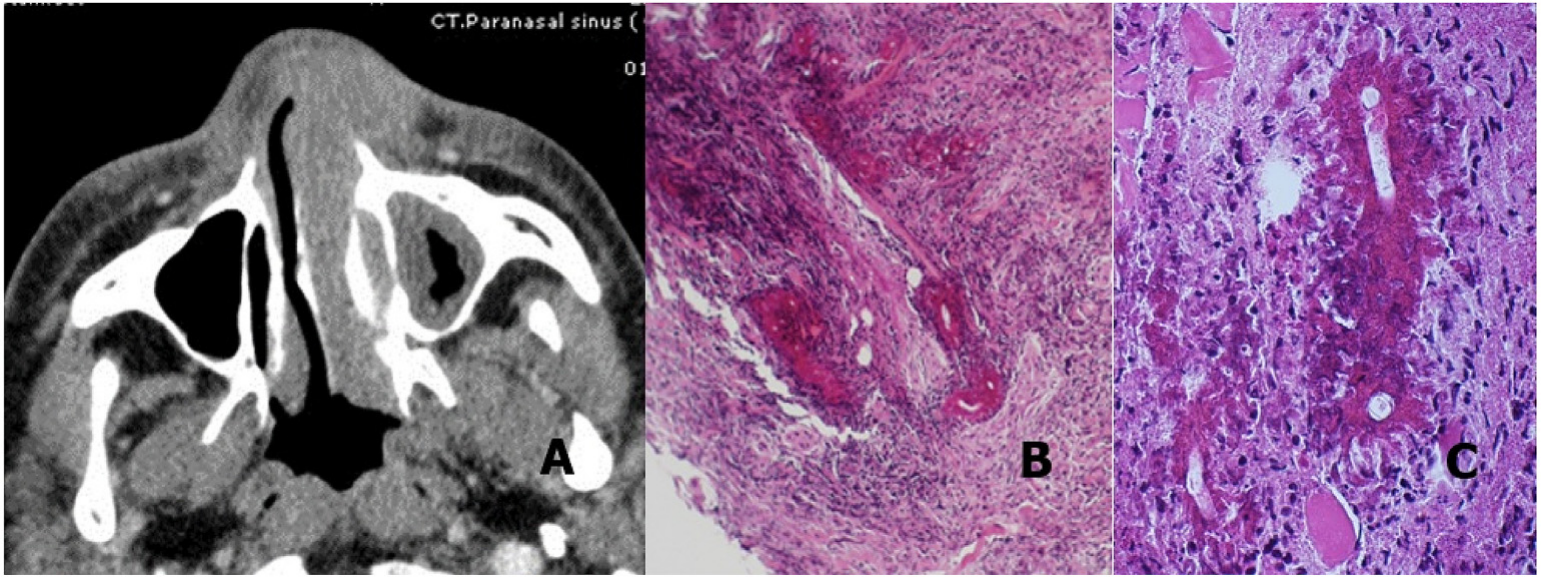
A Axial CT scans showed enhancing soft tissue infiltration over the skin and subcutaneous tissue of left nasal vestibule, inferior turbinate and nasal cavity. The H & E (B) and PAS (C) stains showed large fungal hyphae with surrounding eosinophilic material, chronic inflammation and fibrosis.

Only one patient presented with progressive nasal obstruction without external swelling. Patients had no fever, necrosis, or pus over the skin or in the nasal cavity. The time from disease occurrence to diagnosis ranged from one month to two years. Although the tissue biopsies were performed before sending the patients to the university hospital, the tissue biopsies were performed several times without correct diagnosis. The details of each case are presented in Table 1. The lesions were first suspected of being tumors rather than an infectious process because of their progression and firm consistency. Pathological reports from previous biopsies were acute and chronic inflammation, angiolymphoid hyperplasia with eosinophilia (ALHE), granuloma etc. Computerized tomography (CT) scans showed enhancing soft tissue infiltration in the affected area (See Fig. 2). At our university hospital with Conidiobolomycosis in the differential diagnosis, repeated biopsies were done with a large amount of tissue taken for culture and pathologic examination. In the No. 6 patient who had a preoperative diagnosis of inverted papilloma, intraoperatively the infiltrating lesion was a firm to hard consistency, so the differential diagnosis was changed. The pathological diagnosis of Entomophthoramycosis came from the presentation of large fungal hyphae surrounded with eosinophilic material (Splendore-Hoeppli phenomenon) and eosinophils infiltration in H&E staining. The fungus could be seen better in the periodic acid-Schiff (PAS) and Gomori Methenamine-Silver stain (GMS). Tissue culture showed *Conidiobolus* spp. in two out of seven patients. The No. 4 patient was diagnosed upon clinical presentation, therapeutic response and history of the same disease five years ago [5]. All seven patients responded well after corrected diagnosis and medical treatment. The response was obviously seen within two weeks of treatment, and then the treatment was continued for six months to a year. The protocol of medication varied, but usually contained a combination regimen of itraconazole and potassium iodide (KI)/co-trimoxazole. Itraconazole dosage is 400 mg/day, KI 30 mg/kg/day and co-trimoxazole 2400 mg of sulfamethoxazole/day. The No. 2 and 3 patients have continued the treatment and followed up at their local hospitals. The No. 6 and 7 patients are in the course of treatment. Blood monitoring of side effects was regularly performed every two to three months; thyroid function for KI and liver enzymes for itraconazole were assessed until complete treatment. Two patients (No. 5 and 6) had to stop KI because of hypothyroidism.

**Table 1:** Characteristics of the cases.

No	Age	Year	Occupation	Presenting symptoms	Duration	Physical findings	Underlying disease	Blood eosinophil (%)	Previous pathologic report	Diagnosis	Fungal culture	treatment	Results at last visit
1	57	2009	Barber	persisting nasal obstruction	1 mo	infiltrating lesion at right inferior turbinate	Hypertension	3.2	1. ALHE	pathologic report	NG	KI 2 wk Itraconazole + co-trimoxazole 8 mo	no recurrence at 1 year after complete treatment
2	69	2012	Agriculturist	progressive rhinofacial swelling, nasal obstruction	3 mo 2 mo	firm rhinofacial swelling, infiltrating lesion at nasal vestibule	None	17.9	1. Lymphoid hyperplasia 2. Acute and chronic inflammation 3. ALHE differential diagnosis of lymphoma	pathologic report	NG	Itraconazole	marked improvement at 2 months
3	59	2013	–	progressive rhinofacial swelling, nasal obstruction	3 mo	firm rhinofacial swelling	None	–	1. chronic inflammation with foreign body reaction	pathologic report	NG	Itraconazole	marked improvement at 2.5 months
4	41	2013	Agriculturist 2008 Entomophthoramycosis culture positive of *Conidiobolus coronatus*	nasal obstruction and swelling	2 mo	firm nasal swelling	None	7.6	poorly form granuloma	clinical and therapeutic diagnosis	NG	Co-trimoxazole + Itraconazole 8 mo	completely resolved and no recurrence 1 month after complete treatment
5	47	2018	Worker for Disaster Prevention and Mitigation	nasal obstruction, rhinofacial swelling	2 y 3 mo	firm rhinofacial swelling, infiltrating lesion and nasal vestibule and inferior turbinate	None	5.4	3 times biopsy chronic inflammation Differential diagnosis of lymphoma	pathologic report and culture	Conidiobolus spp.	KI 5 mo + Itraconazole	complete resolve at 10 months of treatment
6	71	2018	–	nasal obstruction, rhinofacial swelling	8 mo 1 mo	Infiltrating lesion and mass in the right nasal cavity	None	22.2	Papilloma	Pathologic report	NG	KI 3 mo + Itraconazole 6.5 mo	complete resolve
7	27	2019	–	epistaxis, nasal swelling	2 mo	nasal swelling and infiltrating lesion at nasal vestibules	congenital HIV infection	17.8	acute and chronic inflammation	pathologic report and culture	Conidiobolus spp.	Itraconazole	improvement at two weeks of treatment

mo: month, ALHE: angiolymphoid hyperplasia with eosinophilia, NG: no growth, KI: Potassium iodide, y; year, wk: week.

## 4 Discussion

The common causes of facial swelling are infection/inflammation such as cellulitis, sinusitis with complications and allergic reactions in acute and chronic forms. The diseases can also be of the skin, of skin appendages and of the subcutaneous tissue or from underlying and surrounding structures such as the nose and paranasal sinuses, orbit, bone, soft tissue, or odontogenic lesion. The tumor can be benign or malignant infiltrations; primary, metastasis, or hematologic, which are less common but must be included in a differential diagnosis. Rhinofacial entomophthoromycosis or Conidiobolomycosis is an uncommon disease, but commonly presents with facial swelling. The disease, once it is known, will not be forgotten. Typically, patients arrive with firmness, indurated skin and subcutaneous tissue over the nasal dorsum, nasolabial area, lip(s), glabella and forehead with or without nasal obstruction. The facial disfiguration progresses slowly, diagnosis is achieved by suspicion of the clinical course and by specific investigation of tissue biopsy and culture. Though this is not really a rare disease, it is under-recognized even in tropical and subtropical areas. In the past, it was called Rhinofacial zygomycosis, under the class Zygomycetes, order Enthomopthorales apart from Mucorales [6]. Because of a different clinical picture, it is now classified in subphylum Entomophthoramycota, which includes Basidiobolomycetes, Neozygitomycetes and Entomophthoramycetes [1]. The typical case of Conidiobolomycosis may occur from spore inhalation or inoculation to the skin under minor trauma in an immunocompetent host, leading to host and pathogen interaction. Patients may have blood eosinophilia, granulomatous, eosinophilic reaction over fungal infection [2], as in our patients. The difficulty of diagnosis occurs when no physicians who encounter it know the disease. The senior dermatologist in our center [7] introduced us to this condition when we first saw the No.4 patient. Since then, Conidiobolomycosis was suspected in the typical presented case and could be diagnosed after one or two biopsies, due to communication between pathologists, otolaryngologists and infectious medicine. Because of the infiltration or mass-like lesions, epithelial and hematologic tumors were in the differential diagnosis. For the marked tissue, eosinophils and chronic inflammation, pathological differential diagnosis was parasitic infestation, Kimura, ALHE, and for the granulomatous formation; foreign body granuloma, fungal infection etc. The high blood eosinophils and Splendore-Hoeppli phenomenon showed the immune reaction between host and fungi, which does not present in Mucormycosis or invasive fungal sinusitis. In these typical cases of Conibolomycosis, there is no need to perform a radical surgery or to provide a toxic antifungal agent such as amphotericin B. The lesions respond well with medical treatment, for example, with azole group in combination with KI or co-trimoxazole as in our patients. Though single agents can be used [1,2], Tondolo et al. studied in vitro and found that the combination of antifungal and co-trimoxazole had a synergistic effect against *Conidiobolus lamprauges* [8]. In the atypical cases that occur in immunocompromised hosts, the disease can spread to the orbit, intracranium or systemic dissemination [2,3]. With those atypical cases, aggressive medical and surgical treatment may be needed. Antifungal reported usage include KI, co-trimoxazole, amphotericin B, ketoconazole, itraconazole, fluconazole, miconazole, voriconazole, terbinafine, and 5-fluorocytosin [3]. The duration of treatment ranged from several months to more than a year [1,3,9]. The facial disfiguration, however, may not completely resolve as the prolonged inflammation may lead to chronic localized fibrosing leukocytoplastic vasculitis [2]. The earlier the diagnosis, the better the result. In our cases, dramatic resolution can be observed within two weeks of medication use. Infiltration over the face and nasal cavity can be cleared in three months, and the continued treatment after that point depends on infectious medicine. There was no recurrence during the follow up period of completed treatment cases. The No. 4 patient was the only one who had a new onset of the disease after five years.

## 5 Conclusions

Rhinofacial entomophthoromycosis or Conidiobolomycosis typically can be diagnosed under the suspicion of a clinical presentation. Pathological tissue, culture and response to treatment confirm the correct diagnosis. The obvious clinical response can be seen within several weeks after medication use. Additional studies for antifungal treatment protocols can be performed with randomized controlled trials.

## Funding

This study is supported by 10.13039/501100002842Faculty of Medicine, Chiang Mai University, Thailand.

## Provenance and peer review

Not commissioned externally peer reviewed.

## Ethical approval

Research Ethics Committee 4, Faculty of Medicine, Chiang Mai University.

Certificate of Approval No.205/2019.

## Consent

Written consent forms were obtained for patients’ photography with Ethics Committee approval.

## Author contribution

Saisawat Chaiyasate: study concept, data collection, data interpretation,writing the paper.

Parichat Salee: study concept, data interpretation, manuscript review.

Kornkanok Sukapan: study concept, data interpretation, writing the paper.

Tanyathorn Teeranoraseth: study concept, data collection, manuscript review.

Kannika Roongrotwattanasiri: study concept, data collection, writing the paper.

## Registration of research studies

1.Name of the registry: Thai Clinical Trial Registry
2.Unique Identifying number or registration ID: TCTR20200608006
3.Hyperlink to your specific registration (must be publicly accessible and will be checked): http://www.clinicaltrials.in.th/index.php?tp=regtrials&menu=trialsearch&smenu=fulltext&task=search&task2=view1&id=6364

## Guarantor

Saisawat Chaiyasate.

## Declaration of competing interest

None.
